# 1,3-Bis(2,6-diisopropyl­phen­yl)-4,5-dihydro-1*H*-imidazol-3-ium triiodide

**DOI:** 10.1107/S1600536810045228

**Published:** 2010-11-10

**Authors:** Monisola I. Ikhile, Muhammad D. Bala

**Affiliations:** a School of Chemistry, University of KwaZulu-Natal, Westville Campus, Private Bag X54001, Durban 4000, South Africa

## Abstract

In the crystal structure of the title compound, C_27_H_39_N_2_^+^·I_3_^−^, the imidazolidinium ring is perpendicular to a mirror plane which bis­ects the cation. The dihedral angle between the imidazolidinium ring and the benzene ring is 89.0 (2)°. The triiodide anion also lies on a mirror plane and is almost linear with an I—I—I bond angle of 178.309 (18)°.

## Related literature

For a related structure with a 1,3-(2,6-diisopropyl­phen­yl)imidazolidinium unit, see: Giffin *et al.* (2010 ▶). For its synthesis, see: Llewellyn *et al.* (2006 ▶).
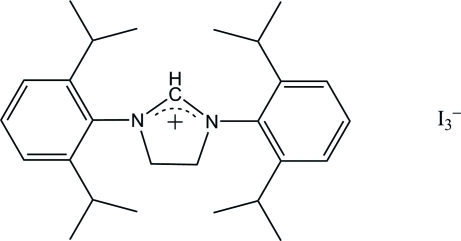


## Experimental

### 

#### Crystal data

C_27_H_39_N_2_^+^·I_3_^−^
*M* *_r_* = 772.30
Monoclinic, 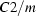

*a* = 18.0288 (5) Å
*b* = 15.4554 (5) Å
*c* = 13.8457 (6) Å
β = 129.456 (1)°
*V* = 2978.81 (18) Å^3^
*Z* = 4
Mo *K*α radiation
μ = 3.16 mm^−1^
*T* = 173 K
0.39 × 0.22 × 0.14 mm

#### Data collection

Bruker APEXII CCD diffractometer
Absorption correction: integration (*XPREP*; Bruker, 2005 ▶) *T*_min_ = 0.438, *T*_max_ = 0.642
12244 measured reflections
3772 independent reflections
2536 reflections with *I* > 2σ(*I*)
*R*_int_ = 0.048

#### Refinement

*R*[*F* ^2^ > 2σ(*F* ^2^)] = 0.039
*wR*(*F* ^2^) = 0.122
*S* = 0.97
3772 reflections
155 parameters
H-atom parameters constrained
Δρ_max_ = 1.92 e Å^−3^
Δρ_min_ = −1.30 e Å^−3^

### 

Data collection: *APEX2* (Bruker, 2005 ▶); cell refinement: *SAINT* (Bruker, 2005 ▶); data reduction: *SAINT*; program(s) used to solve structure: *SHELXS97* (Sheldrick, 2008 ▶); program(s) used to refine structure: *SHELXL97* (Sheldrick, 2008 ▶); molecular graphics: *SHELXTL* (Sheldrick, 2008 ▶); software used to prepare material for publication: *SHELXTL*.

## Supplementary Material

Crystal structure: contains datablocks global, I. DOI: 10.1107/S1600536810045228/is2624sup1.cif

Structure factors: contains datablocks I. DOI: 10.1107/S1600536810045228/is2624Isup2.hkl

Additional supplementary materials:  crystallographic information; 3D view; checkCIF report

## Figures and Tables

**Fig. 1.**
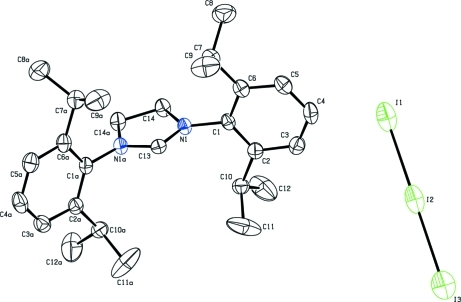
Molecular structure of the title compound with the atom labelling scheme for non-hydrogen atoms. Ellipsoids are drawn at the 50% probability level. All H atoms have been omitted.

## supplementary crystallographic information

### Comment

We were using a general synthetic method that involved the deprotonation of *N*-heterocyclic carbene (NHC) salts with strong bases to generate free carbenes, followed by *in situ* metalation with an iron(II) precursor to generate iron(II) based NHC complexes. In order to obtain piano stool type compounds, η^5^-CpFe(CO)_2_I was used as the iron(II) precursor. Piano-stool type complexes are of interest due to their outstanding spectroscopic and structural features which has made them the subject of many elegant studies in the past. But in this instance, the title compound C_27_H_39_N_2_I_3_, (I), was obtained as a triiodide adduct of the protonated NHC ligand. A molecule of the cationic NHC is characterized by a bisecting mirror plane, while the triiodide counterion is symmetrical around the central iodine atom I2. The imidazolidinium ring is nearly orthogonal to the phenyl rings of the N-substituents with torsion angles N13–N1–C1–C6 close to 90°. The triiodide counterion is linear.

### Experimental

To a suspension of 1,3-bis(2,6-diisopropylphenyl)imidazolidinium chloride (0.1 g) in dry THF (15 ml) was added potassium *tert*-butoxide (0.031 g). After 1 h, this solution was added to a solution of [η^5^-CpFe(CO)_2_I] (0.07 g) in dry toluene (40 ml). After stirring for 20 hrs, the resulting precipitate was centrifuged and washed once with dry toluene (30 ml). The toluene extracts were combined and left standing in air to form shiny black crystals of (I).

### Refinement

Hydrogen atoms were first located in a difference map and then positioned geometrically (C—H = 0.95–1.00 Å) and allowed to ride on their respective parent atoms. The highest peak and the deepest hole in the difference Fourier map are located 0.87 and 0.65 Å, respectively, from atom I3.

### Crystal data

**Table:**

C_27_H_39_N_2_^+^·I_3_^−^	*F*(000) = 1496
*M**_r_* = 772.30	*D*_x_ = 1.722 Mg m^−^^3^
Monoclinic, *C*2/*m*	Mo *K*α radiation, λ = 0.71073 Å
Hall symbol: -C 2y	Cell parameters from 3850 reflections
*a* = 18.0288 (5) Å	θ = 2.9–28.1°
*b* = 15.4554 (5) Å	µ = 3.16 mm^−^^1^
*c* = 13.8457 (6) Å	*T* = 173 K
β = 129.456 (1)°	Block, brown
*V* = 2978.81 (18) Å^3^	0.39 × 0.22 × 0.14 mm
*Z* = 4	

### Data collection

**Table:**

Bruker APEXII CCD diffractometer	3772 independent reflections
Radiation source: fine-focus sealed tube	2536 reflections with *I* > 2σ(*I*)
graphite	*R*_int_ = 0.048
φ and ω scans	θ_max_ = 28.3°, θ_min_ = 1.9°
Absorption correction: integration (*XPREP*; Bruker, 2005)	*h* = −17→24
*T*_min_ = 0.438, *T*_max_ = 0.642	*k* = −20→18
12244 measured reflections	*l* = −18→11

### Refinement

**Table:**

Refinement on *F*^2^	Primary atom site location: structure-invariant direct methods
Least-squares matrix: full	Secondary atom site location: difference Fourier map
*R*[*F*^2^ > 2σ(*F*^2^)] = 0.039	Hydrogen site location: inferred from neighbouring sites
*wR*(*F*^2^) = 0.122	H-atom parameters constrained
*S* = 0.97	*w* = 1/[σ^2^(*F*_o_^2^) + (0.065*P*)^2^ + 5.5612*P*] where *P* = (*F*_o_^2^ + 2*F*_c_^2^)/3
3772 reflections	(Δ/σ)_max_ = 0.026
155 parameters	Δρ_max_ = 1.92 e Å^−^^3^
0 restraints	Δρ_min_ = −1.30 e Å^−^^3^

### Special details

**Table:**

Geometry. All e.s.d.'s (except the e.s.d. in the dihedral angle between two l.s. planes) are estimated using the full covariance matrix. The cell e.s.d.'s are taken into account individually in the estimation of e.s.d.'s in distances, angles and torsion angles; correlations between e.s.d.'s in cell parameters are only used when they are defined by crystal symmetry. An approximate (isotropic) treatment of cell e.s.d.'s is used for estimating e.s.d.'s involving l.s. planes.
Refinement. Refinement of *F*^2^ against ALL reflections. The weighted *R*-factor *wR* and goodness of fit *S* are based on *F*^2^, conventional *R*-factors *R* are based on *F*, with *F* set to zero for negative *F*^2^. The threshold expression of *F*^2^ > σ(*F*^2^) is used only for calculating *R*-factors(gt) *etc*. and is not relevant to the choice of reflections for refinement. *R*-factors based on *F*^2^ are statistically about twice as large as those based on *F*, and *R*- factors based on ALL data will be even larger.

### Fractional atomic coordinates and isotropic or equivalent isotropic displacement parameters (Å^2^)

**Table:**

	*x*	*y*	*z*	*U*_iso_*/*U*_eq_	
C1	0.3529 (2)	0.3426 (2)	0.2270 (3)	0.0250 (7)	
C2	0.3463 (2)	0.3008 (2)	0.3116 (3)	0.0277 (7)	
C3	0.3832 (3)	0.2177 (2)	0.3478 (3)	0.0328 (8)	
H3	0.3808	0.1876	0.4055	0.039*	
C4	0.4236 (3)	0.1775 (2)	0.3015 (4)	0.0388 (9)	
H4	0.4480	0.1203	0.3274	0.047*	
C5	0.4285 (3)	0.2201 (2)	0.2178 (3)	0.0354 (8)	
H5	0.4569	0.1919	0.1873	0.043*	
C6	0.3926 (2)	0.3033 (2)	0.1777 (3)	0.0296 (7)	
C7	0.4002 (3)	0.3505 (2)	0.0879 (3)	0.0332 (8)	
H7	0.3583	0.4031	0.0567	0.040*	
C8	0.3653 (4)	0.2949 (3)	−0.0245 (4)	0.0578 (13)	
H8A	0.3638	0.3297	−0.0849	0.087*	
H8B	0.3006	0.2734	−0.0640	0.087*	
H8C	0.4090	0.2459	0.0029	0.087*	
C9	0.5022 (3)	0.3801 (4)	0.1556 (4)	0.0573 (13)	
H9A	0.5240	0.4145	0.2289	0.086*	
H9B	0.5050	0.4156	0.0992	0.086*	
H9C	0.5440	0.3296	0.1825	0.086*	
C10	0.3020 (3)	0.3437 (2)	0.3629 (3)	0.0310 (8)	
H10	0.2700	0.3981	0.3141	0.037*	
C11	0.3788 (4)	0.3692 (5)	0.4980 (4)	0.080 (2)	
H11A	0.4077	0.3170	0.5497	0.120*	
H11B	0.3500	0.4040	0.5259	0.120*	
H11C	0.4284	0.4031	0.5062	0.120*	
C12	0.2256 (5)	0.2874 (4)	0.3461 (6)	0.0741 (17)	
H12A	0.1784	0.2701	0.2582	0.111*	
H12B	0.1932	0.3200	0.3706	0.111*	
H12C	0.2557	0.2356	0.3986	0.111*	
C13	0.3643 (3)	0.5000	0.2404 (4)	0.0239 (9)	
H13	0.4304	0.5000	0.3122	0.029*	
C14	0.2147 (2)	0.4503 (2)	0.0778 (3)	0.0298 (7)	
H14A	0.1690	0.4272	0.0882	0.036*	
H14B	0.1980	0.4272	−0.0005	0.036*	
N1	0.31536 (19)	0.42912 (17)	0.1873 (2)	0.0250 (6)	
I1	0.24578 (3)	0.0000	0.27734 (5)	0.06328 (17)	
I2	0.34949 (3)	0.0000	0.54335 (4)	0.05203 (15)	
I3	0.45122 (3)	0.0000	0.81697 (5)	0.06143 (17)	

### Atomic displacement parameters (Å^2^)

**Table:**

	*U*^11^	*U*^22^	*U*^33^	*U*^12^	*U*^13^	*U*^23^
C1	0.0219 (16)	0.0203 (15)	0.0278 (14)	−0.0005 (12)	0.0134 (12)	−0.0018 (12)
C2	0.0229 (16)	0.0261 (17)	0.0252 (14)	−0.0019 (13)	0.0111 (13)	−0.0014 (12)
C3	0.0311 (19)	0.0298 (19)	0.0313 (16)	−0.0006 (15)	0.0170 (14)	0.0027 (14)
C4	0.035 (2)	0.0253 (19)	0.045 (2)	0.0043 (16)	0.0201 (17)	0.0037 (15)
C5	0.034 (2)	0.0302 (19)	0.0416 (18)	0.0031 (16)	0.0240 (16)	−0.0041 (15)
C6	0.0221 (17)	0.0294 (18)	0.0328 (15)	−0.0029 (14)	0.0153 (14)	−0.0042 (14)
C7	0.0312 (19)	0.0338 (19)	0.0393 (17)	0.0014 (15)	0.0246 (16)	−0.0014 (15)
C8	0.068 (3)	0.060 (3)	0.038 (2)	−0.019 (3)	0.030 (2)	−0.011 (2)
C9	0.048 (3)	0.081 (4)	0.051 (2)	−0.027 (3)	0.035 (2)	−0.012 (2)
C10	0.0318 (18)	0.0329 (19)	0.0272 (15)	0.0007 (15)	0.0182 (14)	−0.0011 (14)
C11	0.050 (3)	0.115 (5)	0.043 (2)	0.019 (3)	0.015 (2)	−0.035 (3)
C12	0.103 (5)	0.059 (3)	0.118 (5)	−0.025 (3)	0.097 (4)	−0.029 (3)
C13	0.021 (2)	0.026 (2)	0.0233 (19)	0.000	0.0131 (17)	0.000
C14	0.0198 (16)	0.0267 (18)	0.0303 (15)	0.0005 (13)	0.0101 (13)	−0.0013 (13)
N1	0.0201 (13)	0.0214 (14)	0.0267 (12)	0.0006 (11)	0.0118 (11)	0.0002 (10)
I1	0.0473 (3)	0.0391 (3)	0.0848 (3)	0.000	0.0333 (2)	0.000
I2	0.0325 (2)	0.0354 (2)	0.0847 (3)	0.000	0.0356 (2)	0.000
I3	0.0361 (2)	0.0614 (3)	0.0792 (3)	0.000	0.0331 (2)	0.000

### Geometric parameters (Å, °)

**Table:**

C1—C6	1.403 (5)	C9—H9C	0.9800
C1—C2	1.407 (5)	C10—C11	1.509 (5)
C1—N1	1.441 (4)	C10—C12	1.518 (6)
C2—C3	1.386 (5)	C10—H10	1.0000
C2—C10	1.518 (5)	C11—H11A	0.9800
C3—C4	1.384 (5)	C11—H11B	0.9800
C3—H3	0.9500	C11—H11C	0.9800
C4—C5	1.385 (5)	C12—H12A	0.9800
C4—H4	0.9500	C12—H12B	0.9800
C5—C6	1.388 (5)	C12—H12C	0.9800
C5—H5	0.9500	C13—N1	1.302 (3)
C6—C7	1.520 (5)	C13—N1^i^	1.302 (3)
C7—C9	1.511 (5)	C13—H13	0.9500
C7—C8	1.521 (5)	C14—N1	1.484 (4)
C7—H7	1.0000	C14—C14^i^	1.535 (7)
C8—H8A	0.9800	C14—H14A	0.9900
C8—H8B	0.9800	C14—H14B	0.9900
C8—H8C	0.9800	I1—I2	2.8824 (7)
C9—H9A	0.9800	I2—I3	2.9808 (7)
C9—H9B	0.9800		
			
C6—C1—C2	123.1 (3)	H9A—C9—H9C	109.5
C6—C1—N1	118.5 (3)	H9B—C9—H9C	109.5
C2—C1—N1	118.4 (3)	C11—C10—C12	111.6 (4)
C3—C2—C1	116.8 (3)	C11—C10—C2	110.7 (3)
C3—C2—C10	120.7 (3)	C12—C10—C2	112.0 (3)
C1—C2—C10	122.5 (3)	C11—C10—H10	107.5
C4—C3—C2	121.5 (3)	C12—C10—H10	107.5
C4—C3—H3	119.3	C2—C10—H10	107.5
C2—C3—H3	119.3	C10—C11—H11A	109.5
C5—C4—C3	120.4 (3)	C10—C11—H11B	109.5
C5—C4—H4	119.8	H11A—C11—H11B	109.5
C3—C4—H4	119.8	C10—C11—H11C	109.5
C4—C5—C6	120.9 (4)	H11A—C11—H11C	109.5
C4—C5—H5	119.5	H11B—C11—H11C	109.5
C6—C5—H5	119.5	C10—C12—H12A	109.5
C5—C6—C1	117.3 (3)	C10—C12—H12B	109.5
C5—C6—C7	120.7 (3)	H12A—C12—H12B	109.5
C1—C6—C7	121.9 (3)	C10—C12—H12C	109.5
C9—C7—C8	110.7 (3)	H12A—C12—H12C	109.5
C9—C7—C6	110.2 (3)	H12B—C12—H12C	109.5
C8—C7—C6	111.8 (3)	N1—C13—N1^i^	114.6 (4)
C9—C7—H7	108.0	N1—C13—H13	122.7
C8—C7—H7	108.0	N1^i^—C13—H13	122.7
C6—C7—H7	108.0	N1—C14—C14^i^	102.77 (16)
C7—C8—H8A	109.5	N1—C14—H14A	111.2
C7—C8—H8B	109.5	C14^i^—C14—H14A	111.2
H8A—C8—H8B	109.5	N1—C14—H14B	111.2
C7—C8—H8C	109.5	C14^i^—C14—H14B	111.2
H8A—C8—H8C	109.5	H14A—C14—H14B	109.1
H8B—C8—H8C	109.5	C13—N1—C1	125.5 (3)
C7—C9—H9A	109.5	C13—N1—C14	109.9 (3)
C7—C9—H9B	109.5	C1—N1—C14	124.6 (3)
H9A—C9—H9B	109.5	I1—I2—I3	178.309 (18)
C7—C9—H9C	109.5		
			
C6—C1—C2—C3	1.3 (5)	C1—C6—C7—C9	103.2 (4)
N1—C1—C2—C3	−180.0 (3)	C5—C6—C7—C8	49.3 (5)
C6—C1—C2—C10	−179.2 (3)	C1—C6—C7—C8	−133.2 (4)
N1—C1—C2—C10	−0.5 (4)	C3—C2—C10—C11	72.8 (5)
C1—C2—C3—C4	−0.7 (5)	C1—C2—C10—C11	−106.7 (4)
C10—C2—C3—C4	179.8 (3)	C3—C2—C10—C12	−52.4 (5)
C2—C3—C4—C5	0.4 (6)	C1—C2—C10—C12	128.1 (4)
C3—C4—C5—C6	−0.5 (6)	N1^i^—C13—N1—C1	179.3 (2)
C4—C5—C6—C1	1.0 (5)	N1^i^—C13—N1—C14	0.1 (5)
C4—C5—C6—C7	178.7 (3)	C6—C1—N1—C13	−89.2 (4)
C2—C1—C6—C5	−1.4 (5)	C2—C1—N1—C13	92.0 (4)
N1—C1—C6—C5	179.8 (3)	C6—C1—N1—C14	89.9 (4)
C2—C1—C6—C7	−179.1 (3)	C2—C1—N1—C14	−88.9 (4)
N1—C1—C6—C7	2.2 (5)	C14^i^—C14—N1—C13	−0.1 (3)
C5—C6—C7—C9	−74.3 (4)	C14^i^—C14—N1—C1	−179.3 (3)

Symmetry codes: (i) *x*, −*y*+1, *z*.
